# Kaempferol Targets Global Epigenetic Modifiers to Impedes Growth and Migratory Ability of HeLa Cells

**DOI:** 10.1111/jcmm.70498

**Published:** 2025-03-30

**Authors:** Nazia Afroze, Shafiul Haque, Arif Hussain

**Affiliations:** ^1^ School of Life Sciences Manipal Academy of Higher Education Dubai UAE; ^2^ Department of Nursing College of Nursing and Health Sciences, Jazan University Jazan Saudi Arabia; ^3^ School of Medicine Universidad Espiritu Santo Ecuador

**Keywords:** epi‐diet, epigenome, hypermethylation, hypomethylation, kaempferol, methyl sequencing

## Abstract

Dietary bioactive agents can curb tumour progression through chromatin alterations. Thus, this study attempts to evaluate the influence of kaempferol on epigenome modification in HeLa cells. Biochemical analysis for global DNA methylation‐LINE 1, DNMTs (DNA methyltransferases), HAT (histone acetyl transferase), HDACs (histone deacetylases) and HMTs (histone methyltransferases) were examined with their transcript level expression through qPCR. Also, H3 and H4 histone modification marks were quantitated by an ELISA‐based assay. Moreover, qPCR and protein profiler were performed to analyse the expression of migratory genes at both mRNA and protein levels, respectively, that was further substantiated through colony formation, invasion, and scratch wound assays. Finally, DNA methyl‐sequencing was performed to analyse the promoter methylation of TSGs (tumour suppressor genes) and corroborated by analysing selected TSGs' expression. Kaempferol treatment did not alter the global DNA methylation‐LINE 1 compared to untreated control, however, it reduced the expression and biochemical activities of DNMT and HDAC, which can be linked to their hypermethylation by kaempferol exposure. Concordant with the reduced expression of HMTs, HATs and other epi‐enzymes, various histone H3 and H4 marks were also observed to be modulated. Kaempferol exposure led to promoter hypomethylation of various TSGs (such as *WIF1, RUNX1, RARβ, SOX1*), which subsequently led to enhanced expression at the mRNA level, which corresponds to their reactivation. Molecular studies were consistent with cell‐based studies, which demonstrated a strong growth inhibitory and anti‐migratory effect of kaempferol. This research helps to understand the probable mechanism used by kaempferol as a potential epigenetic modifier.

## 1 Introduction

Dietary bioactive agents can curb tumour progression as they can influence the expression of different genes involved in proliferation, migration and invasion, and apoptosis and so forth through modulating DNA methylation and histone modifications [1]. Widespread global DNA hypomethylation, hypermethylation of CpG of promoters, and histone modifications such as methylation, acetylation, phosphorylation and so forth are responsible for heterogeneity in tumour cells along with driving the cells towards carcinogenesis. These modifications alter chromatin structure and limit DNA accessibility to various transcriptional factors essential for gene expression [1]. Hypermethylation at the promoter region of TSGs has been shown to enhance cancer progression by transcriptional repression in various cancers such as p16^INK4a^ involved in cell cycle control, hMLH1 (Human mutL homologue 1) in colorectal carcinoma [2], p53, DAPK (death‐associated protein Kinase), RASSF1 (Ras Association Domain Family Member 1), WIF1 (WNT Inhibitory Factor 1), VHL (von Hippel–Lindau gene), RUNX1 (Runt‐related transcription factor 1), RARβ (Retinoic acid receptor β), *SOX1* [SRY (sex determining region Y)‐box 1] and TIMP (tissue inhibitor of metalloproteinases) in various types of cancers including cervical cancer [3].

Epigenetic modifications are mediated by several enzymes such as DNMTs, DNA demethylases (KDMs), HDACs, HATs, HMTs and phosphorylases, etc. [4, 5]. Deviant manifestations of these enzymes have been confirmed in a variety of carcinomas including cervical carcinoma [2, 6, 7]. Promoter hypermethylation is the key mechanism for epigenetic‐mediated silencing of TSGs due to the enhanced expression of DNMTs. Global hypomethylation of repetitive DNA sequences at CpG sites is the foremost significant consequence for the transformation of a normal cell into a carcinogenic cell [8]. Conversely, DNMT‐impacted hypermethylation of CpG islands of TSG promoters (*Braca1*, *Rb*, or *p53*) causes deactivation of these proteins which consequently drives the cells towards carcinogenesis [8]. DNMTs (DNMT1, DNMT3A and DNMT3B) upregulation is promoted by oncoproteins E6 and E7 and the magnitude of their expression is directly related to cancer progression in CC [9]. Over‐expression of different HDACs (HDAC1, 2, 3 and 6) has been confirmed earlier in different cancers [10]. In many cancers, enhanced HDAC family proteins expression has been reported, including B cell and T cell acute lymphoblastic leukaemia (ALL), indicating the role of histone acetylation in various leukemogenesis [8]. HDAC overexpression in tandem with DNA methylation and other histone modifications such as HMTs, KDMs (Lysine demethylases) and HATs silence tumour suppressor genes [11, 12]. HMTs are responsible for the addition of mono‐, di‐, or tri, ‐CH3 groups onto specific lysine residues. Histone marks on H3 that is, H3K9, H3K27 and H4K20, are vital marks associated with transcription repression whereas H3K4, H3K79, and H3K36 are responsible for transcription activation [6, 7, 13, 14] H3K4me hypomethylation and H3K9me hypermethylation are important for maintaining DNA methylation for gene silencing in colon cancer cells [15]. H4K20me was also reported as an epigenetic marker for several types of cancer cells [16].

Considering the reversible nature of epigenetic alterations, these modifications can be targeted for epigenetic‐based cancer therapy. Epi‐drugs (synthetic epigenetic inhibitors) can curb carcinogenesis. Hatzimichael [17] and Shankar et al. [13] have established that the epigenetic drugs currently under evaluation, that is, DNMT inhibitors (decitabine or azacytidine) or HDAC inhibitors (vorinostat and romidepsin) [12] have shown promising results against solid malignancies [13, 17] however, the narrow specificity and the adverse side effects associated with them warrant drug candidates with a safer profile [13, 18].

Epidemiologic reports have reported that plant‐derived chemopreventive agents/polyphenols can reverse epigenetic modifications of the cancer cells by modulating different epienzymes [19, 20]. Polyphenols such as Quercetin, chrysin, EGCG and myricetinare established to inhibit HDAC and DNMT activities, with a consequent reactivation of TSGs [2, 10, 21] kaempferol (3,5,7‐trihydroxy‐2‐[4‐hydroxyphenyl]‐4*H*‐1‐benzopyran‐4‐one) is a flavonol ubiquitously present in tea, cabbage, kale, strawberry, grapes, and tomatoes [22] Gamut reports have confirmed kaempferol impacted antioxidant, anti‐inflammatory, anti‐neoplastic, and anti‐invasive properties [23, 24]; however, the study on the effect of kaempferol on the epigenome is at its early infancy. Kaempferol is reported to inhibit the action of DNMT3B in bladder cancer and restore the TSG gene DACT2 (Dishevelled Binding Antagonist of Beta Catenin 2) by reducing its methylation via inhibiting DNMT1 directly [25, 26]. Kaempferol inhibited HDAC to inhibit G9a expression to induce autophagy in gastric cancer cells [27]. Another study confirmed kaempferol's inhibitory role on HDAC along with histone H3 hyperacetylation that subsequently deterred proliferation and induced cell death in human colon and hepatocarcinoma [28]. This study intended to investigate the effect of kaempferol on the expression and activities of different chromatin modification enzymes, along with analysing the promoter methylation status of TSGs in HeLa cells.

## 2 Material and Methods

### 2.1 Preparation of Drug Dilutions

A 1 mM stock solution of kaempferol (Cat CAS:520‐18‐3 biosciences) (Tokyo Chemical Industries; Tokyo, Japan) was prepared in DMSO (Dimethyl sulphaoxide) in serum‐free DMEM (Dulbecco's Modified Eagle Medium) media. The purity of the compound is > 97.0% (HPLC). Stocks were aliquoted and placed at −20°C. For treatment, the dilutions were prepared in complete media.

### 2.2 Maintenance of Cell Culture

The model cell line used in this research is the human cervical carcinoma HeLa cell line. It was procured from AddexBio, USA (Cat # C000800). The cell line was maintained in DMEM (enriched with 10% FBS, 1% 100X Pen‐Strep (Sigma, USA), and 2.5 mM glutamine Catalogue number G5792 Sigma Chemical Co., St. Louis, MO, USA) and kept for growth in an incubator having a humidified environment with 5% CO_2_ and a constant temperature of 37°C.

All the assays were performed after treating HeLa cells with kaempferol for 48 h at doses ranging between 30 and 50 μM and some of them at only 50 μM at 48 h. The timeline was selected as 48 h, as the published earlier IC50 of kaempferol was found to be 50 μM at 48 h, so assays were performed either at sub‐lethal doses or at IC50.

### 2.3 Expression Analysis of Selected Genes

To analyse the impact of kaempferol exposure on tumour suppressor and migratory gene transcript expressions in both treated and control cells were performed using TaqMan‐based custom array (4391594). RNA extraction from kaempferol treated (50 μM) and DMSO control was done following the steps mentioned in the GenElute Mammalian Genomic Total RNA Kit (Catalogue No. RTN70 Sigma‐Aldrich, Merck KGaA) followed by cDNA synthesis as described previously [29]. The qPCR was carried out in QuantStudio3 followed by analysis through ^ΔΔ^CT method. The relative expression or fold change (FC) was computed through the DataAssist program in comparison to the DMSO control. Data was normalised using GAPDH as the housekeeping gene. Statistical significance was established at *p* ≤ 0.05.

### 2.4 Analysis of Different Chromatin Modification Enzymes

The effect of kaempferol (50 μM) at 48 h on chromatin modifying enzymes was done at the transcript level. RNA extraction after kaempferol exposure and control HeLa cells was done as mentioned earlier. Manifestation of various chromatin modification enzymes was assessed by RT^2^ Profiler PCR Array Human Epigenetic Chromatin Modification Enzymes (Cat No. PAHS‐085Z; Qiagen, USA). Also, 1 μg of cDNA was diluted with nuclease‐free water. Then 25 μL of a mix of cDNA and qPCR master mix (Catalogue number: 330504; Qiagen, USA) in a 1:1 ratio was added to each well with the coated primers. Then the plate was subjected to run on Applied Biosystem; QuantStudio 3 (USA). Data were normalised through GUSB as a housekeeping gene followed by fold change calculation, and the relative expression was calculated in comparison to DMSO control. Graphpad Prism version 9.3.1 was used to show the data significance.

### 2.5 DNMT Activity Assay

DNMT enzyme is not associated only with gene silencing; rather, it has an important role in normal mammalian development, and its aberrant expression is correlated with various diseases, including cancer. Hence, the impact of kaempferol on DNMT activity was assessed to analyse the anti‐carcinogenic activity of kaempferol. The assay was performed using the DNMT Inhibition/Activity Quantification Kit (abcam, cat no. ab113467, UK) following the protocol provided. Nuclear extract was prepared following the steps as per the EpiQuikTM Nuclear Extraction Kit (Catalogue No. OP‐0002, Epigentek, USA) from both untreated and DMSO control HeLa cells [2]. The assay was completed following the steps mentioned in the kit. Briefly, 30, 40, and 50 μM of kaempferol solutions were made in DNMT buffer. A 50 μL mix of nuclear extract, DNMT buffer, drug solution, and Adomet was added to the substrate‐coated wells and allowed to undergo enzymatic reaction. Later, the capture antibody was added, followed by the detection antibody. Then, the signal was developed, followed by O.D. (Optical Density) measurement by ELISA at 450 nm. % enzyme inhibition was calculated compared to control using the formula below.

$$\begin{array}{cc} \text{Inhibition}\%= & [1–\text{inhibition sample}\;\mathrm{OD}–\text{Blank}\;\mathrm{OD}/\;\\ & \mathrm{No}\text{inhibition sample}\;\mathrm{OD}–\text{Blank}\;\mathrm{OD}]\times 100 \end{array}$$

### 2.6 HDAC Activity Assay

HAT and HDAC enzymes cause acetylation and deacetylation of the chromatin that subsequently leads to euchromatinisation and heterochromatinisation. Hence, the HDAC Activity Assay Kit (BioVision; Cat no. K 331, Cambridge) was used to investigate the consequence of kaempferol on HDAC activity. Kaempferol solutions with nuclear extract were added to each well followed by HDAC buffer and substrate (which contains acetylated lysine side chain). Then the plate was kept at 37°C for 60 min to allow HDAC of the sample to deacetylate the substrate. After which, the signal was developed by incubating the plate with a lysine developer for 30 min. Finally, readings were taken through ELISA at 405 nm. The inhibitory effect of kaempferol on HDAC was calculated compared to the control sample (without kaempferol) and the result was plotted as % inhibition in the graph.

### 2.7 HAT Activity

The prepared nuclear extract, as mentioned above, was used to analyse the effect of kaempferol on histone acetyltransferase activity HAT Activity/Inhibition Assay Kit (BioVision; Cat no. K 332, Cambridge) following the steps given in the kit. Briefly, the assay mix was prepared, comprising assay buffer, HAT substrates I and II, and NADH‐generating enzymes. In three wells, the assay mix was added with only nuclear extract, while in others, the drug was also added at 30, 40 and 50 μM followed by incubation for 3 h. Finally, after the colour development, O.D. reading was taken. HMT H3K27 inhibition assay.

### 2.8 HMT H3K27 Methyltransferase Activity

The HMT H3K27 Methyltransferase Activity/inhibition Quantification Assay Kit (abcam, cat no. ab113454, UK) was procured to measure the impact of kaempferol on HMT H3K27 activity. The assay was completed following the steps in the kit's protocol. Briefly, a mix of nuclear extract assay buffer, biotinylated substrate, drug concentration alongside and Adomet (methyl group donor) was added to each coated well and kept for 60 min at 37°C. The plate was incubated on an orbital shaker for 30 min with the Capture antibody. Finally, the plate was incubated with the detection antibody, followed by the addition of developing solution to develop colour. After the colour development (blue), the reaction was stopped, followed by measuring the O.D. reading at 450 nm. The percentage of enzyme inhibition was compared with the sample without kaempferol and presented as a bar graph.

### 2.9 Global DNA Methylation Assay‐LINE 1

DNA isolation was performed following the steps of the GenElute Mammalian Genomic DNA Miniprep Kit (Catalogue No. G1N70, Sigma‐Aldrich, Merck, KGaA) kit. Evaluation of alteration in the methylation status of repeated sequence after kaempferol (30–50 μM) in comparison to untreated control sample was done by the Global DNA methylation LINE‐1 kit (Catalogue number: 55017; Active Motif, USA). Briefly, an equal amount of each concentration of quantitated DNA sample was subjected to enzymatic digestion followed by hybridisation with a biotinylated probe. Immobilisation of the hybridised DNA was done in the coated plate. Then, 5mC antibody alongside HRP‐streptavidin labelled secondary antibody was employed to develop colour. Reading was taken by a microplate reader at 450 nm. Absorbance value of the treated sample was compared to assay control to calculate comparative LINE‐1 methylation level. Data are shown as an average of SD (±) of three experiments.

### 2.10 Evaluation of Histone H3 and H4 Modifications

To evaluate kaempferol impacted modification of histone H3 and H4 modification marks, the histone extraction kit (cat. No. ab113476) and H3 and H4 modification multiplex assay kit (Catalogue Nos. ab185910 and ab185914, abcam, UK) were acquired. Around ~100 ng of extracted histone was added to each coated well. The volume of each well was made up to 50 μL that is, 46 μL of antibody buffer and 4 μL of histone extract (treated/untreated for control) followed by incubation at 37°C for 2 h. Then, the detection antibody was added, followed by the developer solution. After the colour development, the stop solution was supplemented in each well. Absorbance was read, and calculation was done to demonstrate the effect of treatment as a percentage on various histone marks. For all the biochemical assays, significance was established at *p* ≤ 0.05, and a two‐way ANOVA was used.

### 2.11 Clonogenic Assay

The principle of the colony formation assay is based on the capability of a few live cells to produce colonies. Approximately ~4 × 10^5^ cells/well plated cells were exposed to kaempferol (30, 40, and 50 μM). After 48 h, both treated and control cells were collected, and around 500 cells/well were allowed to grow. The steps were replicated as per the protocol published earlier [2]. The experiments were repeated for reproducibility. To establish significance at *p* ≤ 0.05, two‐way ANOVA was used.

### 2.12 Impact of Kaempferol on Cell Migration

To understand if kaempferol can impact wound healing, an assay was done. The protocol was followed as per the earlier published paper from our lab [30]. Briefly, about ~3 × 10^5^ cells/well were plated in 12 wells. On a confluent monolayer, a wound was dragged using a 200 μL tip. Increasing concentrations of kaempferol (30, 40, and 50 μM) were used for treatment. Cell migration was captured at 0 h and then at an interval of every 24 h until wound closure. Measurement of the wound width was done from the captured images through MS Paint, and the % of wound closure was shown as a bar graph. Significance is established at *p* ≤ 0.05 through One‐way ANOVA.

### 2.13 Invasion Assay

The invasion assay evaluated the migratory and invasive capabilities of kaempferol‐treated HeLa cells versus a DMSO control using the Boyden chamber [2]. HeLa cells (5.0 × 10^3^ cells/well) were seeded in the upper chambers of a 24‐well plate [2], with FBS‐containing medium in the lower wells. After 48 h, cells were fixed with methanol and stained with 0.1% crystal violet. Non‐invading cells were removed. Migration was assessed using an inverted microscope at 200× magnification, with images captured for analysis.

### 2.14 Quantitation of Protein Expression of Migration Related Genes and TGFβ Pathway

The analysis of the anti‐migratory impact of kaempferol manifestation of 10 different proteins involved in migrations was evaluated by Human MMP antibody array (Abcam; Cat no. ab134004, UK) and TGFβ pathway RayBio were analysed. Differential expression of these proteins was analysed through kit protocol. Concisely, ~1.2 × 10^6^ cells per flask were plated, followed by kaempferol treatment (50 μM at 48 h). The rest of the steps were followed as published previously [22] The differential expression level was done by quantitating the intensity of the dot blot on treated and control membranes through Image Lab software (version 6.1). Then relative quantitation of the treated sample was done after normalising with the reference spot. Significance was established at *p* ≤ 0.05.

### 2.15 Quantitation of Promoter Methylation Through Methyl Sequencing

To detect DNA CpG methylation in HeLa cells and its alteration after exposure to the dietary agent kaempferol, methyl‐sequencing was performed. After isolation of DNA, the purification and quantitation of the isolated DNA were done through a nanodrop spectrophotometer (Thermo Scientific; 2000).

#### 2.15.1 Library Prep

Approximately, 500 ng of isolated DNA from control and treated cells after shearing to produce fragments with a peak size of 150–200 bp, followed by checking the fragment length using Agilent TapeStation (Agilent Technologies, Palo Alto, CA, USA). Then the fragments were subjected to end‐repair, adenylation and ligation with Illumina adaptors. Further, the fragments with the adaptors attached were purified, followed by hybridisation and finally eluted. The isolated fragments were exposed to bisulfite conversion utilising the EZ DNA Methylation Gold Kit, which leads to the conversion of unmethylated C to U residues while the methylated Cs do not undergo any modification cytosine. Then the amplification of the DNA fragments was done through PCR (8 cycles) which leads to the conversion of U to T residues, followed by clean‐up of the products with JetSeq Beads. The modified libraries were then placed for indexing, PCR amplification, followed by clean‐up.

#### 2.15.2 Illumina Sequencing

The libraries were then subjected to sequencing for 150 cycles on the Illumina HiSeq X Ten sequencer (Illumina, San Diego, USA) according to the kit's protocol, generating 40–54 million reads per sample.

#### 2.15.3 Bioinformatics Analysis

The raw Illumina reads from control and kampferol (30 μM) treated Human samples were subjected to quality check using the FastQC v0.11.3^2^ tool. The high‐quality processed reads were obtained using Trimgalore v0.4.0^3^ tool. The processed reads were used for performing bismark alignment against human genome version hg19. The alignments were further used for the Bismark methylation extraction. The extracted CpG context was used for the identification of DMRs using methy1Kit^5^ (R package). Annotation for the CpG context was performed using Homer. Methylation differences > 0.10 with *p*‐values < 0.05 were considered significant.

Heap map was generated using the R package–gplots to show the differential methylation pattern between control and kaempferol (20 μM) treated Hela cells. All genes' names were compiled for the functions of interest (Biological Process); however, only the top 40 genes were used as input for network generation using the ShinyGO tool. PPI network using STRING in the background and network analysis for biological process functions were generated.

### 2.16 Statistical Analysis

All the experimental data are shown as an average ± standard deviation of three experiments. GraphPad prism (version 9.3.1) was used to compute all experiment results. Significance was established at *p* ≤ 0.05 through two‐way ANOVA followed by Tukey's HSD post hoc analysis.

## 3 Results

### 3.1 Kaempferol Attenuated DNMT and HDAC Activities

**FIGURE 1:**
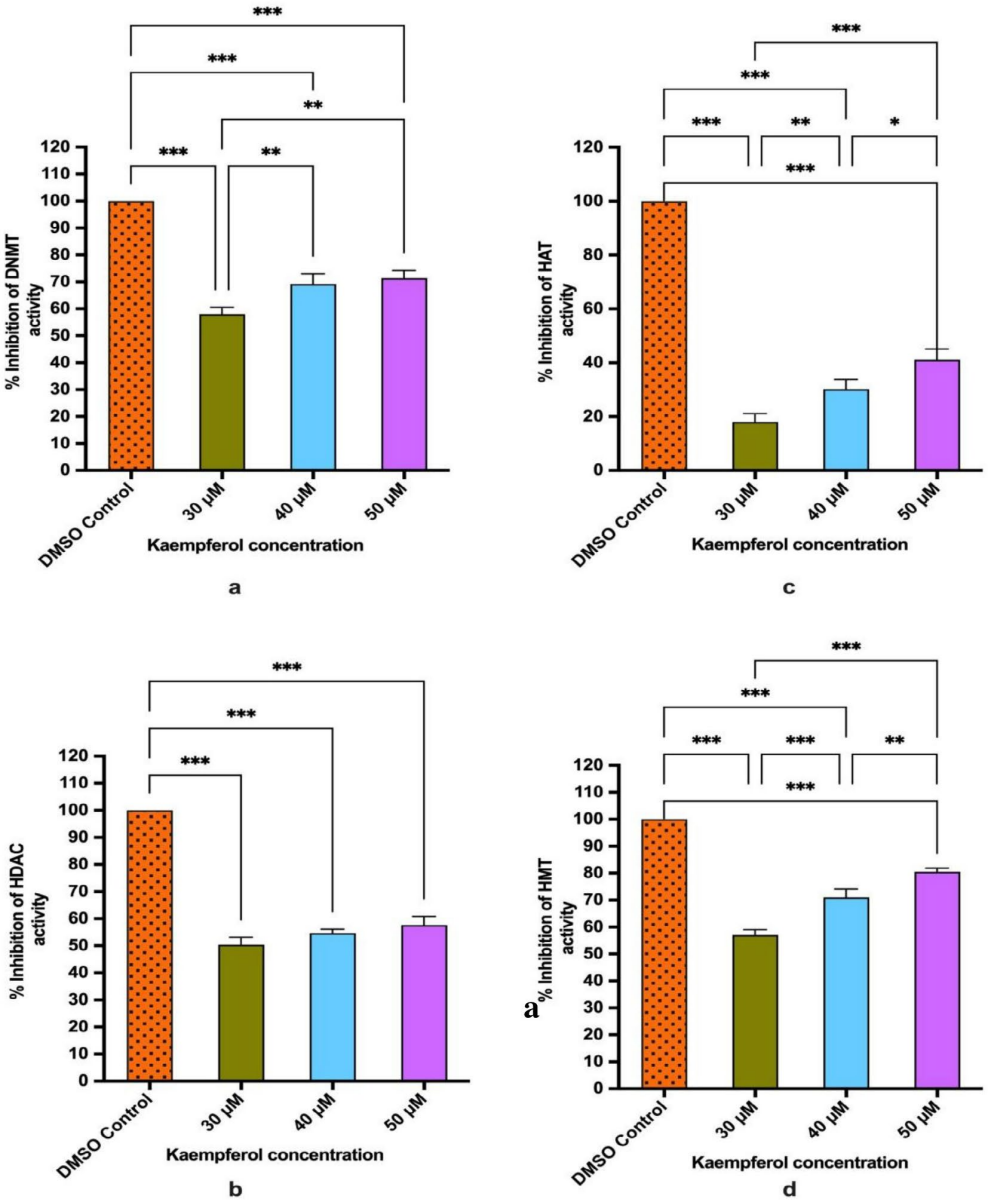
(a) Bar graph showing kaempferol impacted % inhibition of DNMT activity at 48 h. (b) Bar graph showing dose dependent inhibition of HDAC activity by kaempferol treatment at 48 h. (c) Bar graph showing kaempferol treatment (48 h) reduced of HAT activity. (d) Bar graph representing kaempferol impacted % inhibition of HMT H3K27 activity. Data are presented as the mean ± standard deviation of three independent experiments. Significance was established at *p* ≥ 0.05. Two way‐ANOVA *p < 0.05, **p < 0.01, ***p < 0.001.

DNMT and HDAC activities were demonstrated to be downregulated by kaempferol compared to control in a concentration‐dependent mode. Kaempferol exposure of 30, 40 and 50 μM (48 h) was observed to inhibit DNMT activity from 100% (DMSO control) to 58.4%, 69.2%, and 71.6%, respectively (Figure 1a). Similarly, kaempferol also repressed the HDAC activity by 53%, 55%, and 57% compared to untreated control (Figure 1b). Data are presented as the mean ± standard deviation of three independent experiments (Figure 1b).

### 3.2 Kaempferol Reduced HAT (Histone Acetyl Transferases) Activity

HAT causes acetylation of histone proteins. Kaempferol (30, 40 and 50 μM) reduced the HAT activity in a dose‐dependent manner by 18%, 30% and 41% respectively in comparison to control (Figure 1c).

### 3.3 Kaempferol Inhibited Biological Activity of HMT That Causes Methylation of H3 at K27

Histone methyl transferase causes methylation (mono, di or tri‐methylation) of H3 at lysine 27, a repressive transcriptional mark. Methylation brought about by HMT on H3K27 with other epigenetic modifications may cause silencing of various TSGs; therefore, their inhibition may subsequently lead to reactivation of these silenced tumour suppressor genes. The % enzyme inhibition of nuclear extract with Kaempferol (30, 40 and 50 μM) was increased in concentration‐dependent manner by 57%, 71% and 82%, respectively, compared to DMSO control (Figure 1d).

### 3.4 Kaempferol Modified the Expression of Chromatin Modifying Genes

**FIGURE 2:**
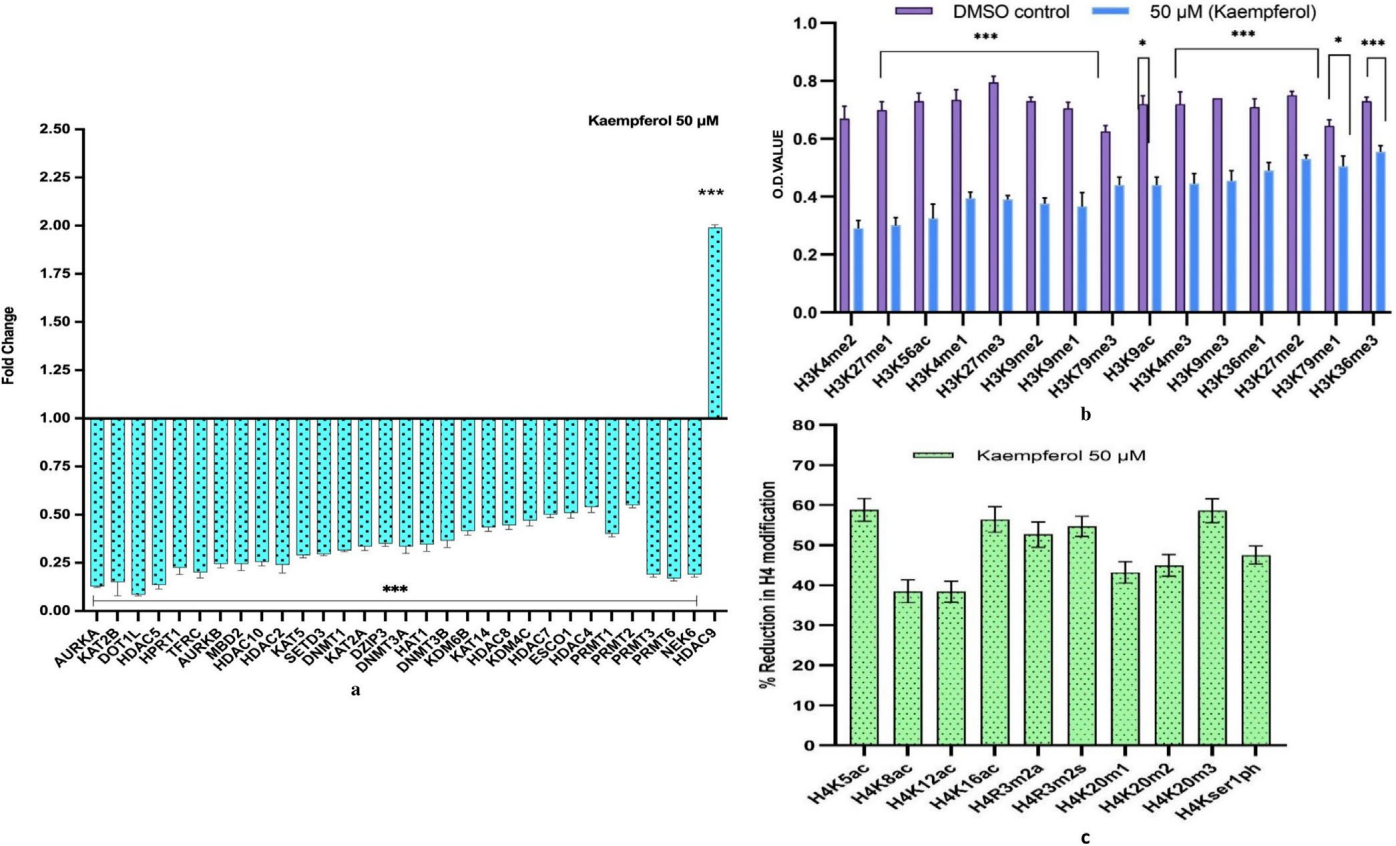
Chromatin modifier expression analysis: (a) Kaempferol treatment at 50 μM for 48 h demonstrated to modify various chromatin modification enzyme compared to untreated control. (b) Histone H3 marks modifications: Kaempferol treatment at 50 μM for 48 h exhibited to decrease various Histone H3 modification marks. (c) Histone H4 marks modification: Kaempferol treatment at 50 μM for 48 h exhibited to decrease various Histone H3 modification marks respectively. *p < 0.05, ***p < 0.001.

Kaempferol downregulated the expression of several chromatin modifying enzymes, which include DNMT1, DNMT3A, DNMT3B, HDAC2, 4, 5, 7 and 10 and histone acetyl transferase (HAT 1), HDAC 9 expression was found to be elevated. Additionally, decreased expression of acetyl transferases (KAT5, 14, 2A), phosphorylases (AURKA, AURKB), acetyl transferase ESCO1 and NEK6 (serine/threonine kinase) DOTL1, and KDM6B (lysine demethylase) were observed after kaempferol treatment with 50 μM at 48 h on HeLa cells (Figure 2a).

### 3.5 Kaempferol Modulated All the H3 and H4 Histone Marks

Kaempferol treatment repressed histone H3 and H4 marks. The methylation on histone H3 at K4 (mono, bi and trimethylation), H3K9 (mono, bi and trimethylation), H3K27 (mono, bi and trimethylation), H3K36 (mono, and trimethylation), and H3K79 (mono, and trimethylation) marks were reduced with kaempferol treatment (50 μM) for 48 h. Moreover, the acetylation marks on histones 3 and 4, such as H3K9ac, H3K56ac, H4K5ac, H4K8ac, H4K12ac and H4K16ac were also found to be reduced after treatment. Different Phosphorylation marks such as H4ser10 p, H3ser28p, H4R3m2a and H4Rm2s were also decreased after 50 μM kaempferol treatment (Figure 2b,c).

### 3.6 Kaempferol Treatment Enhanced the Expression of TSGs at the mRNA Level

**FIGURE 3:**
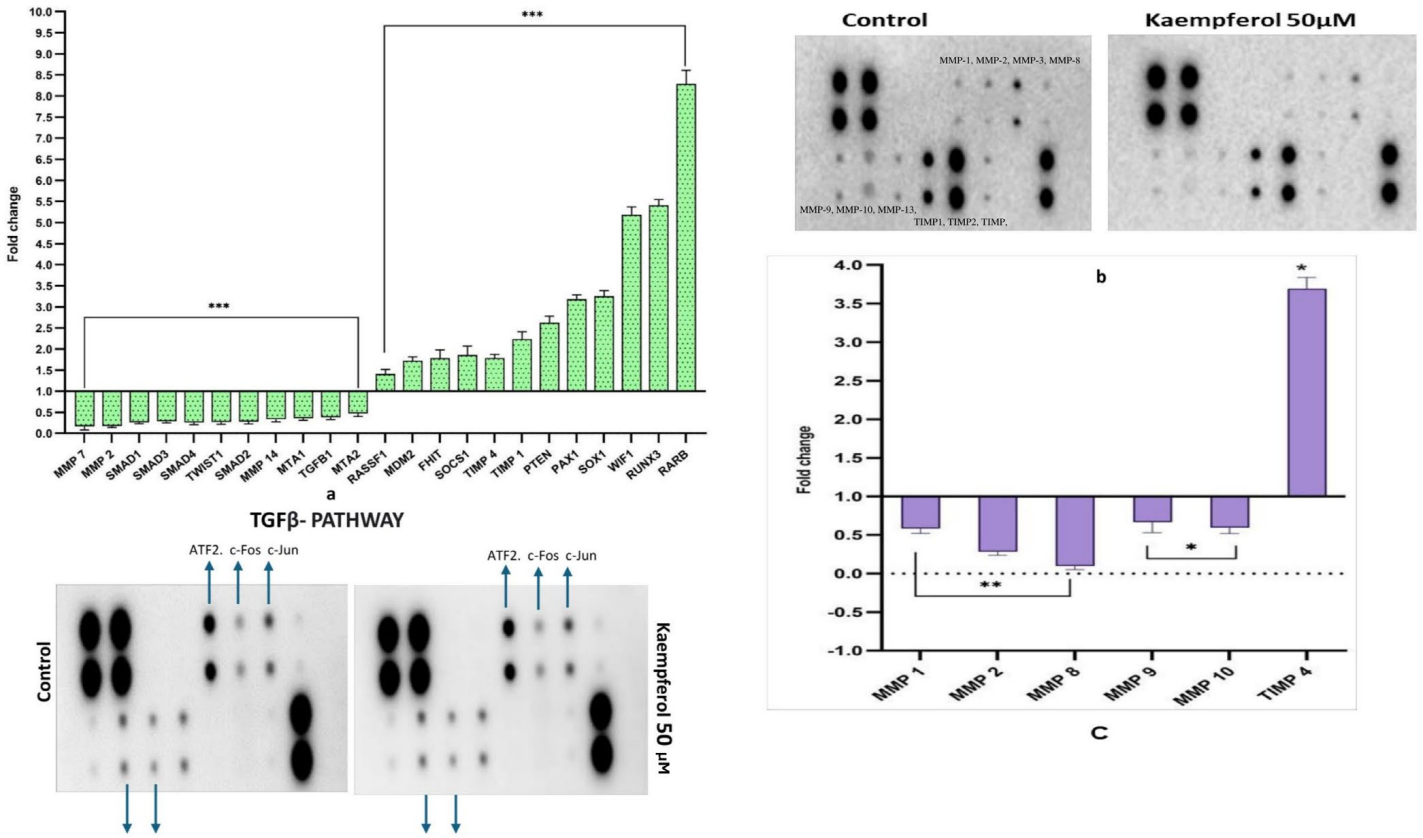
Transcript expression: (a) Bar graph showing qPCR result of kaempferol mediated upregulation of various TSGs expression with a simultaneous downregulation of different oncogenes mainly involved in cell migration with respect to DMSO control HeLa cells. ***p < 0.001. (b) Differential expression of migratory proteins: Images of nitrocellulose membrane and graph representing reduced expression of various MMPs while increased in TIMPs expression followed by kaempferol treatment at 48 h respectively, compared to control. *p < 0.05, **p < 0.01. (c) Images of nitrocellulose membranes showing altered fold change of different phosphorylated proteins involved in TGFB pathways after kaempferol (50 μM of for 48 h) compared to control.

To confirm kaempferol impacted transcriptional elevation owing to promoter demethylation of TSGs, qRT‐PCR was carried out for the selected TSGs. kaempferol 50 μM treatment upregulated the expression of RASSF1 (FC‐1.5), MDM2 (FC‐1.65), FHIT (FC‐1.6), SOCS1 (FC‐1.7), TIMP1 (FC‐2.11), TIMP4 (FC‐1.63), PTEN (FC‐2.51), PAX1 (FC‐3.1), SOX1 (FC‐3.11), WIF1 (FC‐5.01), RUNX3 (FC‐5.47), RARβ (FC‐8.46) and CDH1 (FC‐1.9). Interestingly, kaempferol treatment downregulated the expression of MMP2 (FC‐0.14) and TWIST1 (FC‐0.26) (Figure 3a).

### 3.7 Kaempferol Suppresses the Expression of Genes Associated With Migration at Both Transcript and Protein Levels

At mRNA level, the WNT/TG β pathway was significantly suppressed by kaempferol, as demonstrated by the reduced manifestation of *WNT1* (FC; 0.18), *WNT2* (FC; 0.15), *SMAD1* (FC; 0.24), *SMAD2* (FC; 0.27), *SMAD3* (FC; 0.31), *SMAD4* (FC; 0.25) and *TGFβ1* (FC; 0.37) transcripts, along with genes involved in migration and invasion such as *MMP 2* (FC; 0.14), *MMP7* (FC; 0.09), *MMP14* (FC; 0.33), *MTA1* (FC; 0.36), *MTA2* (FC; 0.48) and *TWIST* (FC; 0.26) while *TIMP1* (FC; 2.11) and TIMP4 (FC; 1.71) were observed to be upregulated (Figure 3a). Consistently, at protein level also genes involved in migration and invasion were also modulated. Kaempferol suppressed migratory genes such as MMP1 (0.54), MMP2 (FC; 0.31) and MMP8 (FC; 0.1), MMP9 (FC; 0.56) and MMP10 (FC; 0.5) whereas TIMP‐4 (FC; 1.7) inhibitors of MMPs, were overexpressed compared to control (Figure 3b). Further, suppression of TGFβ by both agents was mediated by dephosphorylating SMAD2 (P‐Ser245/250/255) (FC; 0.07) and SMAD4 (P‐Thr277) (FC; 0.1) whereas the phosphorylation of ATF2 (P‐Thr69/71) was increased compared to untreated HeLa cells to produce their anti‐migratory activity (Figure 3c).

### 3.8 Kaempferol Deterred Colony Formation, Suppressed Wound Healing and Migratory Ability of Hela Cells

**FIGURE 4:**
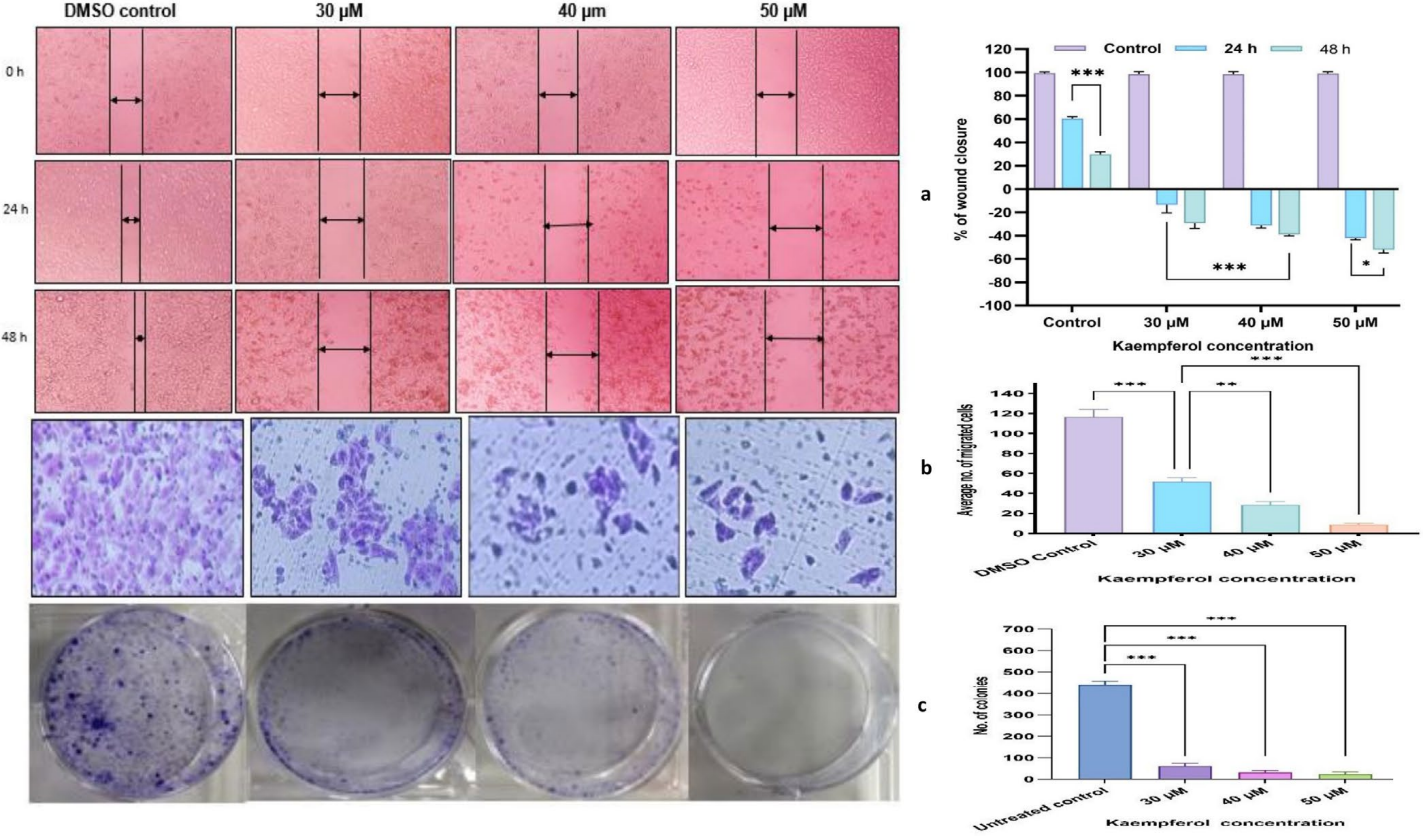
Mitigation of migratory capacity of HeLa cells: (a, b) Kaempferol (30, 40 and 50 μM) treated cells illustrated a significantly increased wound width with increasing time and concentration dependent mode while in trans well assay depicted a sharp reduction in number of migrated Hela cells in contrast to control. (c) Clonogenic assay: Colony assay and bar graph showing the control and kaempferol (30, 40, and 50 μM) treated HeLa cells after 2 weeks of growth. *p < 0.05, **p < 0.01, ***p < 0.001.

Kaempferol treatment in wound healing and migration assay demonstrated a reduction in the wound healing capacity and migratory ability of Hela cells in transwell assay in a concentration‐dependent manner compared to the untreated control. Kaempferol was found to widen the wound width from 16% to ~31% and 21% to 43% at 24 and 48 h respectively between 30 and 50 μM (Figure 4a), whereas complete closure of the scratch in DMSO control was observed after 72 h. Similarly, kaempferol treatment reduced the proportion of migrated cells from ~40% to ~8% between 30 and 50 μM compared to the DMSO control cells (Figure 4b).

Further, kaempferol 30, 40 and 50 μM exposure reduced both the number and dimensions of colonies formed in a dose‐dependent manner when compared to untreated cells; therefore, demonstrating the anti‐proliferative property. Untreated HeLa cells exhibited a survival factor of ~95%, while the number of colonies showed a steep decline to 90, 73 and very few colonies at 30, 40 and 50 μM (Figure 4c). Therefore, this proves the cytostatic property alongside apoptosis‐inducing property of kaempferol.

### 3.9 Kaempferol Modulated the Methylation Status of the Different Genes but Did Not Induce any Alteration to Global DNA Methylation LINE 1

**FIGURE 5:**
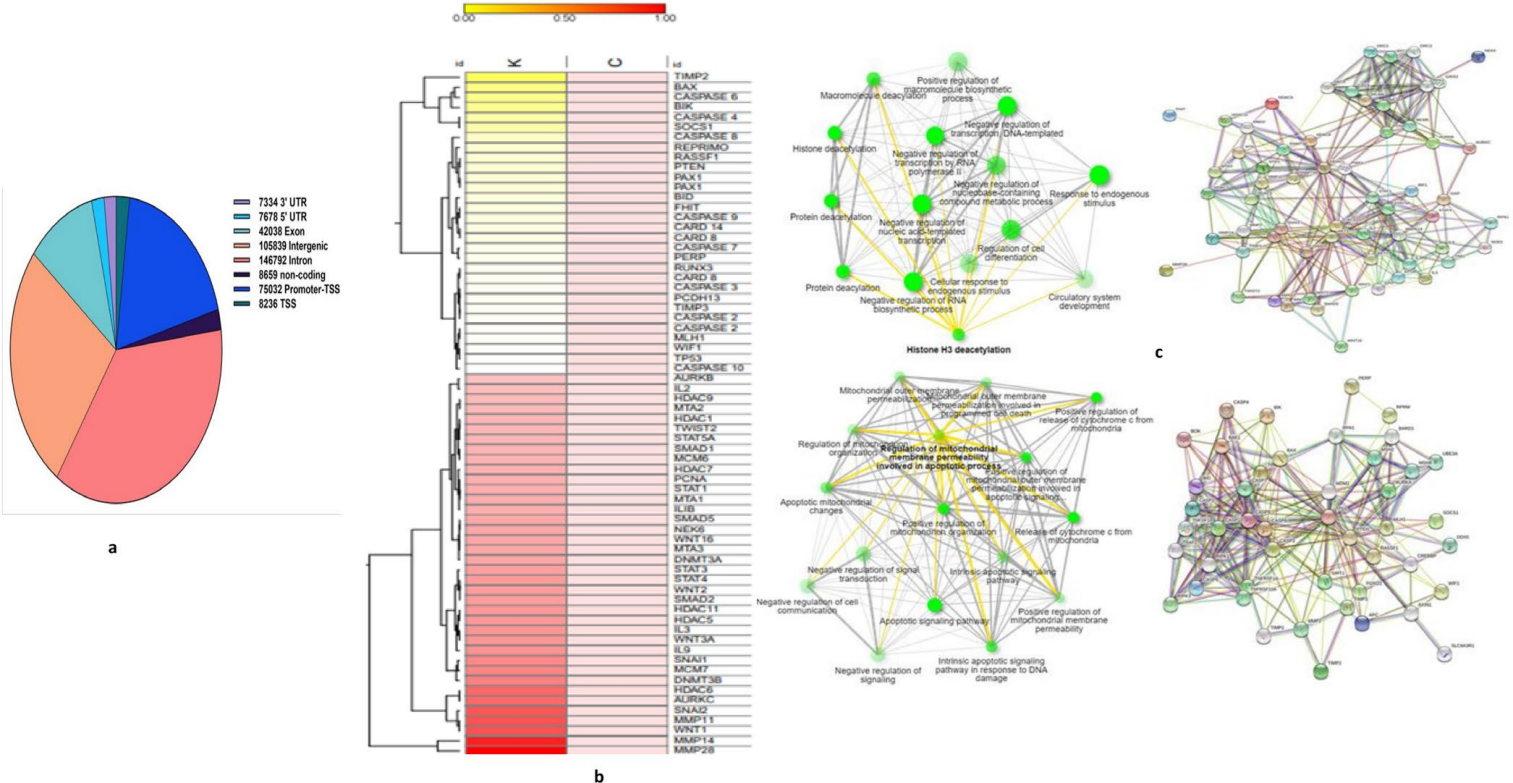
(a) Venn diagram showing distribution of annotation of differentially methylated regions (DMRs) by gene features. (b) Heat map showing the fold change of difference in methylation percentage of various genes between control and kaempferol treated samples (20 μM at 48 h). (c) Showing PPI network and GO‐biological process showing interaction between different hypermethylated gene of interest and showing PPI network and GO‐biological process of hypomethylated genes as an impact of kaempferol treatment on HeLa cells.

The alteration in the methylation level between treated and control groups was analysed to understand kaempferol's effect on methylation. Each sample was run in duplicates, followed by analysis and finally the two files were merged to identify DMR (differentially methylated regions). Finally, the average of the two was considered for presenting the data. The cutoff of 10% was considered to identify the DMRs. The annotated DMRs found in kaempferol treated samples irrespective of the location compared to control are shown in the pie chart (Figure 5a).

Heat map showing differential methylation at the promoter of only the top 70 genes between control and kaempferol‐treated samples (Figure 5b). Notably, kaempferol treatment led to the hypomethylation and reactivation of different TSGs such as PAX1 (25.3%), PTEN (26%), RASSF1 (26%), RUNX3 (15.6%), SOCS1 (41%), SOX1 (19.3%), WIF1 (13%), MDM2 (16.4%), TIMP2 (83.5%), PCDH1 (21.3%), PCDH13 (14.75%) and so forth as their methylation percentage was reduced compared to control. Whereas genes such as MCM6 (11.7%), MCM7 (31.9%), SMAD1 (11.7%), SMAD2 (23.8%), SMAD5 (19.74%), MMP 11 (49.35%), MMP 14 (66.67%), MMP 28 (78.57%), COX2 (12.5%), COX18 (12.7%), MTA2 (13.8%), MTA3 (20.2%), PCNA (17.4%), HDAC 5 (25.2%), HDAC 6 (41.7%), HDAC 7 (16.8%), HDAC 9 (13.8%), HDAC 11 (25.01%), NEK6 (19.8%), SNAI1 (30.7%), SNAI2 (48.14%), AURKB (11.5%), AURKC (41.91%), WNT3A (26.2%), WNT16 (20%) and TWIST2 (14.9%) were found to be hypermethylated; therefore, their methylation percentage was increased, as evident through differential methylation (Figure 5b).

Notably, kaempferol (30, 40 and 50 μM) treatment did not show any alteration in the methylation level of LINE 1 elements compared to untreated control, which clearly indicates its specificity.

All genes' names were compiled for the functions of interest; however, only selected genes were used as input for Biological Process using the ShinyGO tool and for PPI network generation using STRING. Network analysis for the biological generated process shows kaempferol's influence on cell proliferation, cell–cell signalling, and protein phosphorylation besides other physiological processes (Figure 5c).

## 4 Discussion

Dietary bioactive compounds, have the perspective to impede tumorigenesis through regulating the manifestation of genes involved in different cancer hallmarks such as cell proliferation, apoptosis, migration and invasion. These alterations are achieved through DNA methylation and histone modifications [1]. Key mechanisms include global DNA hypomethylation, CpG promoter hypermethylation, and various histone modifications such as methylation, acetylation, and phosphorylation. Together, these epigenetic alteration leads to chromatin modification structure and limit DNA accessibility to various transcriptional factors essential for gene expression which subsequently promotes carcinogenesis [31, 32]. Promoter [1] hypermethylation TSGs can enhance cancer progression by transcriptional repression in various cancers such as *p16* ^*INK4a*^ involved in cell cycle control, *hMLH1* in colorectal carcinoma [2], *p53, DAPK, RASSF1, WIF1, RUNX1, RARβ, SOCS1, SOX1, TIMP* in various types of cancers including cervical cancer [3, 33, 34]. Besides *c‐myc* (myelocytomatosis oncogen), *H‐Ras* (*Harvey rat sarcoma viral oncogene homologue*), *N‐Ras* (*Neuroblastoma rat sarcoma viral oncogene homologue*) and *K‐Ras* (*Kirsten rat sarcoma viral oncogene homologue*), and *CDKs* (*cyclin dependent kinases*), cyclins, have prominent role in the progression of CC as their expression was found to be levated in CC, and some of these genes cause poor prognosis [2, 35].

Epigenetic modification can be targeted for epigenetic‐based cancer therapy due to its reversible nature [8]. Synthetic epigenetic inhibitors (such as decitabine or azacytidine/vorinostat and romidepsin) have shown promising results against solid malignancies in impeding cancer progression [12, 13, 36, 37, 38, 39] however, the limited specificity and the undesired side effects lead to a quest for safer drug candidates [13, 18].

This clearly demonstrates the necessity of using effective chemo‐preventive agents that can effectively reverse epigenetic modifications with extraordinary specificity, thus making them an imperative therapeutic target to be considered. Earlier studies have established that polyphenols modify various signalling pathways to curb migration and modulate epigenetic mechanisms to reverse carcinogenesis [10, 21]. Considering the safer profile and epigenetic modifying capacity of kaempferol, its effect on epigenetic modification was studied on HeLa cells. An important influence on tumorigenesis is aberrant methylation of promoter regions of TSGs by DNMTs leading to their inactivation, which is recognised along with histone modification as a critical mechanism involved in oncogenesis [31]. Overexpressed DNMT in CC increases DNA methylation of the 5′ CpG promoter, which may lead to gene silencing [9, 40]. Notably, kaempferol at 30, 40 and 50 μM (48 h) was observed to reduce DNMT activity in a dose‐dependent manner (Figure 1a). Kaempferol reduced the activity of HMT that methylates histone H3 at lysine 27 which is a repressive transcriptional mark, from 57% to 82% at 30, 40 and 50 μM concentrations compared to DMSO control (Figure 1d). In line with this study, kaempferol was found to reduce DNMT1, DNMT3B and HDAC activity in different cancers [25, 26]. Consistently, at the mRNA level, DNMT1, 3A and 3B were found to be significantly reduced by kaempferol to a FC ≤ 0.5 (Figure 2a). DNMT1 proteins are stabilised by PI3K/AKT and WNT pathways, thus aiding its activity [41]. Kaempferol is reported to regress PI3K/AKT and WNT pathways, therefore indirectly contributing to reduced DNMT activity. Reduced manifestation and activity of DNMT promote reactivation of TSGs, which in turn regulate both proliferation and apoptosis [14, 31]. Various polyphenols have been reported to reduce the expression of DNMTs in different in vitro and in vivo cancer model [2, 10, 42]. HDACs (HDAC1, 2, 3 and 6) overexpression has been confirmed earlier in different cancers [2]. Non‐histone proteins may also get deacetylated by HDACs such as TP53, hence rendering them inactive [2]. Histone modifications and HDAC overexpression, in tandem with DNA methylation, cumulatively silence TSGs [11]. Interestingly, kaempferol treatment repressed HDAC activity in HeLa compared to DMSO control (Figure 1b). The reduction in biochemical activity is well correlated with transcript level reduction in fold change for HDAC 2, HDAC 4, HDAC 7 and HDAC 10; however, HDAC 9 was increased in fold change (Figure 2a). Downregulation of HDAC reactivates TSGs, deters proliferation, and reduces cell survival [12].

HAT1, which is important for achieving clonogenicity and is frequently upregulated in many cancers, including HeLa cells [43]. kaempferol (30, 40 and 50 μM) reduced the HAT activity in a dose‐dependent manner (Figure 1c). The altered expression of HAT supports the anti‐proliferative activity of kaempferol, which was evident as kaempferol reduced the number of colonies in a dose‐dependent manner (Figure 4c). Acetyltransferase such as KAT2A is found to be involved in oncogenesis, and it is responsible for acetylating H3K9, along with other non‐histone acetylations that contribute to cancer progression [44]. Notably, kaempferol was observed to reduce the expression of KAT2A (FC; 0.22) along with reducing the histone H3K9 mono‐, di‐, and trimethylation marks. While KAT2B could not cross the cutoff of 0.6 folds (Figure 2a). Further, a decrease in different acetylation marks on H4 was observed by kaempferol, such as H4K5, K12 and K16 acetylation (Figure 4c) which is consistent with the reduced activity of HAT at biochemical and transcript studies. Polyphenols have exhibited similar results in different cell lines, including HeLa cells [2]. Additionally, methylation (mono‐, di and tri‐methylation) marks on H4K20 and phosphorylation marks on H4Kser1p were found to be reduced by kaempferol at 50 μM (Figure 2c). The observed reduction in these histone marks is in concordance with the reduced transcript expression of AUR kinases (Figure 4a).

HMTs can methylate histone H3 and H4 proteins by adding up to three methyl groups at specific lysine residues. Methylation marks such as H3K4, H3K36, and H3K79 facilitate transcription; hence, they are considered active marks, whereas HMT H3K20 and H3K27 act as repressive transcriptional marks [2]. Methylation brought about by HMT on H3K27, along with other epigenetic modifications, may cause silencing of various TSGs; therefore, their inhibition may subsequently lead to reactivation of these silenced TSGs [45]. An extensive HMT enzyme inhibition was observed, influenced by kaempferol. Kaempferol inhibited the enzyme activity by 82% at the highest tested dose (Figure 1d) along with suppression of these histone 3 marks (Figure 2b). In line with this study, a similar reduction in these histone H3 marks was observed by chrysin [2]. This study showed consistent downregulation of the expression of various Histone H3 marks, along with DNMT, HDAC, HAT, and HMT in qPCR, which was well correlated with the reduction of enzyme activity (biochemical level), along with the reduction of phosphorylated HDAC 2 (FC; 0.42) and 4 (FC; 0.47) by kaempferol (Figure 2a).

Histone kinases catalyse histone phosphorylation and are linked with altered rates of transcription, apoptosis, DNA damage and chromatin condensation during mitosis and meiosis [46]. AURKA A, B, and NEK 6 aid in tumorigenesis by increasing proliferation, cell cycle passage through the G2‐M checkpoint, invasion and metastasis, and these genes are upregulated in cervical cancer [46]. Expression of all three genes is significantly downregulated by both agents. Reduced NEK6 drives the cells towards apoptosis. A similar result has been reported in colorectal carcinoma [47]. DOTI/KMT4 plays a significant role in cell growth and angiogenesis, and hence its reduction by kaempferol justifies the anti‐carcinogenic property (Figure 2a,b). Kaempferol reduced ESCO1 expression (FC ≥ 0.6), which promotes cell survival and proliferation following DNA damage [10]. Perhaps kaempferol's impact on cell death induction and cell cycle arrest is due to induced DNA damage (Figure 2a).

PRMTs (Protein arginine methyltransferases) add a methyl group to various histone and some non‐histone proteins at arginine residues, affecting numerous cellular activities. Upregulation of PRMTs in cancer causes silencing of TSGs; thus, it makes them a significant therapeutic target [10]. PRMT 6 directly targets and methylates promoters of p21 and p53; therefore, reduced expression of PRMT 6 arrests the cell cycle at G2 with the concomitant upregulation of p21 and p27 [10]. PRMTs 1, 3, 5, and 6 were all downregulated by kaempferol. A similar result was reported in the human osteosarcoma cell line (U2OS) [48]. Histone KDM6B (lysine demethylase) and KDM4C are found to be higher in cervical carcinoma [49]. KDM6B is responsible for colony formation ability while KDM4C increases cancer aggressiveness, respectively [50]. Therefore, it is noteworthy that kaempferol treatment has been shown to reduce the expression of both KDM6B and KDM4C, which signifies their growth‐regressing property (Figure 2a).

The functional outcome of modulating DNMT enzymes can be estimated based on global DNA methylation, gene‐specific promoter methylation and expression of tumour suppressor genes. Hypomethylation of LINE 1 is correlated with carcinogenesis [2, 51] Notably, kaempferol did not show any considerable alteration in the global methylation level of LINE 1 at increasing concentrations; therefore, it exhibited a differential action and a safe profile. Hyper‐and hypomethylation of the promoter region of TSGs result from enhanced and reduced activity and expression of DNMT enzymes respectively [31]. A substantial therapeutic advancement in the treatment of cancer can be achieved by reversing the gene specific 5′ promoter CpG methylation [52], and methyl sequencing studies confirmed that kaempferol at a tested dose of 30 μM (48 h) has shown a significant reduction in 5′ CpG promoter methylation and consequent reactivation of TSGs such as *TP53, DAPK1, PAX2, PAX1, PAX6, PTEN (PINK1), RASSF1, WIF1, VHL, RUNX1, RUNX2, RARβ, SOCS1, SOX1, FOXO3, PERP, TIMP 2, TIMP 3, APC2, PCDH1, CDH13* and so forth compared to control (Figure 5a). Most of the said TSGs are found to be suppressed in cervical cancer [53, 54].

DAPK1, a serine/threonine protein kinase, is involved in endorsing apoptosis [10]. TSG genes are found to be suppressed in cervical cancer [53, 54]. Kaempferol mediated restoration of various TSGs was supported by qPCR results, which showed an enhanced expression of selected TSGs such as *RARβ, RASSF1, TIMP3, TP53, PTEN*, etc. (Figure 3a). PTEN prevents cell proliferation and cell migration. PTEN's impact on cell growth is reported to be deterred by suppressing MAPK phosphorylation [10]. RARβ regulates cell proliferation; its promoter methylation is positively correlated with cervical disease grade [2]. HeLa cells showed a similar response of demethylation and re‐expression of RARβ gene when treated with quercetin and chrysin [2, 10]. SOCS1 (Suppressor of cytokine signalling‐1) a negative regulator of JAK–STAT signalling, acts via increasing Rb protein, which subsequently regresses cell proliferation [55]. RASSF1A and FHIT are involved in regulating the cell cycle and apoptotic cell death alongside other cellular functions [10]. WIF1 functions as an inhibitor of the WNT pathway and is thus able to impact proliferation, migration and apoptosis [16]. SOX1 is a key gene associated with the Wnt pathway. Kaempferol has been reported to have a role in reversing methylation [54]. TIMP2 and TIMP3 are inhibitors of MMPs, linked with mitigating migration and invasion, and are reported to be inactivated through promoter hypermethylation in CC [15]. It also regulates the expression of various genes, including HIF1α [56]. Kaempferol reduced TIMP2 and TIMP3 methylation (5B). APC codes for a TSG that induces apoptotic death and antagonises the WNT signalling pathway by inhibiting the migration of cancer cells [10]. Therefore, kaempferol‐mediated reduced methylation of APC2 to 63% indicates its role as a suppressor of the Wnt pathway. Remarkably, this is the very first study reporting the effect of kaempferol on the modulation of different epigenetic mechanisms and the reduction of methylation with the subsequent restoration of different TSGs such as *TP53, PTEN (PINK1), RASSF1, WIF1, VHL, RUNX1, RUNX2, RARβ, SOCS1, SOX1, FOXO3*, and so forth which are otherwise reported to be inactivated through hypermethylation in different cancers [34] (Figures 3a and 5b).

EMT can begin when any of the EMT inducers, such as Twist, TGF‐β1 or Snail, are overexpressed because it increases the expression of FOXC2 [52, 57, 58] As cancer progresses, there is a noticeable cadherin flip from E‐ to N‐cadherin. E‐cadherin (CDH1), responsible for cell–cell adhesion [59, 60]. CDH1 activity is inhibited by SNAILS. Vimentin, Snails, TWIST1, MTA1 and MTA2 promote metastasis [61] TGFβ/SMAD signalling is linked to invasion and migration; hence, its suppression downregulates the expression of MMP and TWIST 1 [52]. MMPs, promotes tumour invasiveness and metastasis by destroying the ECM proteins; whereas, ROBO/TIMPs antagonise its effect by blocking the catalytic action of MMP [62]. Therefore, the equilibrium between MMPs and TIMPs is critical to maintain cell to cell contact [62]. TIMP1, TIMP2 and TIMP3 inactivation is reported in CC patients [62]. Interestingly, kaempferol was observed to suppress TGFβ/SMAD signalling at both mRNA and protein levels by downregulating SMADs, WNT1 and WNT2, TWIST, MMPs, and TGFβ1; whereas, CDH1, TIMP1 (FC; 1.5), and TIMP2, etc. (Figure 3a). In lung carcinoma and melanoma cell lines, kaempferol was found to inhibit cell viability and migration by inhibiting the WNT pathway [10]. Inhibition of MTA1 and TGF‐β/ Smad reverses invasion and metastasis in cervical cancer [63]. Earlier, kaempferol was shown to exhibit an anti‐migratory effect in osteo and lung carcinoma [64, 65]. TGF‐Β1 impacted inhibition of phospho‐smad2/3 in thyroid carcinoma after curcumin treatment is linked with the suppression of EMT [66]. Interestingly, in this study, kaempferol was observed to reduce the expression of TGFβ1. Further, suppression of TGFβ pathway was mediated by SMAD4 (P‐Thr277) and SMAD5 (P‐Ser463/465) in comparison to untreated HeLa cells to produce their anti‐migratory activity (Figure 3c). Phosphorylation of human c‐Jun or Thr69/71 from ATF2 by MAPKs leads to enhanced AP‐1 transcriptional activity [67]. The results of molecular level studies were consistent with cell‐based assays as kaempferol (30, 40, and 50 μM) was observed to mitigate migration and invasion of HeLa cells in both concentration‐and time dependent modes (Figure 4a,b) Similar results from various polyphenols have been reported earlier [29]. Based on the results, it can be inferred that the restoration of tumour‐suppressor gene functions plays a crucial role in the anticancer potential of kaempferol.

## 5 Conclusion

Kaempferol was observed to have great potential as an epigenetic modifier, as it significantly altered the expression of different chromatin modifiers such as DNMTs, HDACs, HAT and HMT and correlatively hypomethylated various TSGs in HeLa cells. The transcript level expression results concordantly reflected at the enzymatic level, as biochemical assays showed that these agents lowered the activity of HAT, DNMTs, HDACs and HMTs in a dose‐dependent manner, which could probably be owing to the inhibition of the enzymes. Kaempferol showed a safe profile, as global DNA methylation of LINE 1 levels was unaltered in treated cells. Based on the epigenetic changes, kaempferol treatment negatively regulated cell proliferation and migration of Hela Cells. Further, in vitro and in vivo studies are warranted to validate the efficacy of kaempferol on epigenetic modulation in cancer cells.

## Author Contributions

**Nazia Afroze:** data curation (equal), formal analysis (equal), investigation (equal), methodology (equal), writing – original draft (equal). **Shafiul Haque:** validation (equal), visualization (equal), writing – review and editing (equal). **Arif Hussain:** conceptualization (equal), data curation (equal), formal analysis (equal), funding acquisition (equal).

## Ethics Statement

The study was approved by the Institutional Review Board.

## Conflicts of Interest

The authors declare no conflicts of interest.

## Data Availability

No, authors do not have any research data outside the submitted manuscript file.
